# An Experimental Investigation of the Behavior of Strain-Hardening Cement-Based Composites (SHCC) under Impact Compression and Shear Loading

**DOI:** 10.3390/ma13204514

**Published:** 2020-10-12

**Authors:** Ali A. Heravi, Oliver Mosig, Ahmed Tawfik, Manfred Curbach, Viktor Mechtcherine

**Affiliations:** 1 Institute of Construction Materials, TU Dresden, 01062 Dresden, Germany; ahmed.tawfik@tu-dresden.de (A.T.); mechtcherine@tu-dresden.de (V.M.); 2 Institute of Concrete Structures, TU Dresden, 01062 Dresden, Germany; oliver.mosig@tu-dresden.de (O.M.); manfred.curbach@tu-dresden.de (M.C.)

**Keywords:** impact loading, split-Hopkinson bar, SHCC, ECC, compressive loading, shear loading

## Abstract

The ductile behavior of strain-hardening cement-based composites (SHCC) under direct tensile load makes them promising solutions in applications where high energy dissipation is needed, such as in earthquakes, impacts by projectiles, or blasts. However, the superior tensile ductility of SHCC due to multiple cracking does not necessarily point to compressive and shear ductility. As an effort to characterize the behavior of SHCC under impact compressive and shear loading relevant to the aforementioned high-speed loading scenarios, the paper at hand studies the performance of a particular SHCC and its constituent, cement-based matrices using the split-Hopkinson bar method. For compression experiments, cylindrical specimens with a length-to-diameter ratio (l/d) of 1.6 were used. The selected length of the sample led to similar failure modes under quasi-static and impact loading conditions, necessary to a reliable comparison of the observed compressive strengths. The impact experiments were performed in a split-Hopkinson pressure bar (SHPB) at a strain rate that reached 110 s^−1^ at the moment of failure. For shear experiments, a special adapter was developed for a split-Hopkinson tension bar (SHTB). The adapter enabled impact shear experiments to be performed on planar specimens using the tensile wave generated in the SHTB. Results showed dynamic increase factors (DIF) of 2.3 and 2.0 for compressive and shear strength of SHCC, respectively. As compared to the non-reinforced constituent matrix, the absolute value of the compressive strength was lower for the SHCC. Contrarily, under shear loading, the SHCC demonstrated higher shear strength than the non-reinforced matrix.

## 1. Introduction

The low energy absorption capacity of concrete makes it vulnerable to dynamic loading. Depending on the type of structure, dynamic loads can be generated by various sources such as traffic and naturally originated dynamic loads, e.g., rock-fall impact and wind-borne debris impact [1,2]. In an effort to improve ductility and energy absorption capacity, fibers have been used in cement-based materials as reinforcement. The promising performance of the composites that were produced triggered extensive research on various types of fiber-reinforced concrete (FRC) [3]. Among the innovative short-fiber reinforced cement-based composites, strain-hardening cement-based composites (SHCC) also known as engineered cement-based composites (ECC), comprise a type of composite capable of reaching higher stress levels after formation of the first crack under tensile loading. This strain-hardening behavior is accompanied by the extensive formation of multiple fine cracks perpendicular to the loading direction [4,5]. With its high energy absorption capacity, such a material can be used in strengthening existing structural elements against extreme loading conditions, e.g., impact and blast loading [6].

To design and optimize the strengthening/protective layers or structural elements made of SHCC, proper characterization of this material under impact loading is required. Such a loading condition corresponds to the range of strain rates higher than 1 s^−1^ [7,8]. Depending on the stiffness, shape, and speed of the impactor, various strain rates can be generated in structures subject to dynamic loading. Thus, impact experiments at various strain rates are required to describe material behavior over the entire range of possible strain rates. The split-Hopkinson bar setup is the most common and reliable testing technique for characterizing material at high strain rates, while the strain rates of approximately 100 s^−1^ are commonly applied in such material characterization. Extensive research is available on the behavior of various types of FRC tested in the split Hopkinson pressure bar (SHPB) [3]. An increase in the compressive strength at high strain rates has been coherently reported regardless of the fiber type. However, when the compressive strength of FRC at high strain rates is compared to that of a non-reinforced constituent matrix, the available results are inconsistent. A reduction in compressive strength was reported in [9] for adding straight steel fibers to a high-strength concrete. By contrast, a slight increase was reported in [10] for using spiral steel fibers in a normal-strength concrete and also in [11] for the addition of straight steel fibers to a high-strength concrete. These results indicate the importance of the fiber and matrix types with respect to the behavior of cement-based composites under impact compressive load.

Regarding the FRC made with polymeric fibers, especially strain-hardening, cement-based composites, fewer research data are available. The behavior of different normal-strength SHCC compositions made of polyvinyl-alcohol (PVA) fibers and granulated blast furnace slag as supplementary cementitious material was studied in [12]. A maximum dynamic increase factor (DIF) of 1.5 has been reported for the SHCC mixtures investigated at a strain rate of 140 s^−1^. Moreover, the performance of a specific SHCC made with steel and PE fibers was studied in [13] at different strain rates. A dynamic increase factor of 1.7 was reported at a strain rate of approximately 100 s^−1^. Although the studies performed provided valuable insights into the behavior of various SHCCs under impact compressive loading, a comparison with the constituent matrix was not reported. Such a comparison could reveal the influence of fibers and their orientation on compressive behavior at high strain rates. Furthermore, the quasi-static experiments performed to derive DIF in the studies mentioned above were performed on specimens with different geometries than those tested in the SHPB. In quasi-static tests, cylindrical specimens usually have an hourglass failure pattern caused by the friction at their ends. However, as a result of tensile stress created on the outer layer of the specimen, under impact loading the failure is caused by numerous cracks parallel to the loading direction [9,10,11,12,13]. Such a failure pattern indicates the confinement of the inner core by the outer layer of the specimen [14,15]. Hence, the different geometries and failure modes of the specimens tested under quasi-static and impact loadings may significantly influence the obtained dynamic increase factors.

For shear loading, several researchers have studied the positive influence of fibers on the resistance of concrete [16,17,18]. However, limited data are available on the strain rate sensitivity of FRC. A DIF of 1.6 for the shear strength of the ultra-high performance concrete (UHPC) reinforced with 1.5% steel fibers was reported in [19]. The complexity of the testing setup and the challenges related to obtaining shear failure in impact experiments are the main reasons behind the very low number of studies on impact shear behavior of fiber-reinforced cement-based materials.

This paper reports an experimental study of the compressive behavior of SHCC and its constituent non-reinforced matrix under impact loading using a SHPB. Two SHCC mixtures under investigation previously showed favorable behavior under impact tensile loading [20,21]. The same normal-strength cement-based matrix is used for both SHCC mixtures. However, one of them is made with high-density polyethylene (HDPE) fibers of 6 mm length and the other with fibers of 12 mm length. By comparing the results of SHCC to those of the non-reinforced matrix, the influence of the fibers on the compressive behavior is investigated. Moreover, based on the failure modes obtained in specimens of two different lengths, the influence of this parameter on the specimen’s failure mode and the compressive behavior obtained is discussed. Accordingly, for both the quasi-static and impact experiments, cylindrical specimens of the same geometry are used. The selected geometry results in similar failure modes in the specimens tested under impact and quasi-static loading. Digital image correlation (DIC) is used to monitor the damage of the specimen during the test.

Moreover, the behavior of the SHCC mixture made with the 6 mm HDPE fibers and the non-reinforced constituent matrix are studied under impact shear loading. For this purpose, an adapter for performing impact shear experiments in a SHTB was developed. Details of the test setup are described. An appropriate measurement technique, which takes into account the influence of the adapter on the wave analysis in SHTB, is suggested and used. Moreover, using high-speed imaging and the subsequent DIC analysis, the failure of the specimen is monitored. Finally, the condition for shear failure in the specimen is discussed.

## 2. Experimental Program

### 2.1. Materials under Investigation

Both SHCC mixtures under investigation were made of the same normal-strength matrix containing cement, fly ash and fine sand; Table 1 provides the mixture compositions. We used 2% by volume fractions of HDPE fibers with two different lengths, 6 and 12 mm to produce the strain-hardening composites. Properties of the HDPE fibers are presented in Table 2. Since the plain matrix was named M1 in the previous investigations [20,21], the same name is used in this study. The SHCC mixtures made of 6 and 12 mm PE fibers are denoted as M1-6PE and M1-12PE, respectively.

For the compression experiments, the mixtures were produced in an Eirich mixer (Germany) with a capacity of 30 L and subsequently cast in beams with dimensions of 100 mm × 100 mm × 750 mm. One beam was cast per mixture. Since relatively small specimens were needed for shear tests, a Hobart mixer with a capacity of 5 L was used for mixing. Three prisms with dimensions of 160 mm × 40 mm × 40 mm were cast for each mixture. It should be noted here that the casting was performed layer by layer to prevent trapping large air bubbles. Note as well that the filling method of the moulds defined a dominant fiber orientation in the longitudinal direction of the moulds.

### 2.2. Setup for Impact Compression Tests

**Figure 1:**
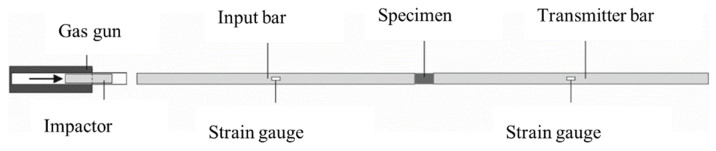
Schematic view of the split-Hopkinson pressure bar (SHPB) [22].

Compression experiments at high strain rates were performed using a SHPB; see Figure 1. The setup consists of an input bar and a transmitter bar with a length of 2.75 m and a diameter of 50 mm, each made of aluminum with a Young’s modulus of 72 GPa. A gas gun accelerates a 130 mm long striker made of brass to a velocity of 17 m/s. The striker hits the input bar and generates a pressure wave that propagates almost one-dimensionally along the bar at the speed of sound in aluminum.

**Figure 2:**
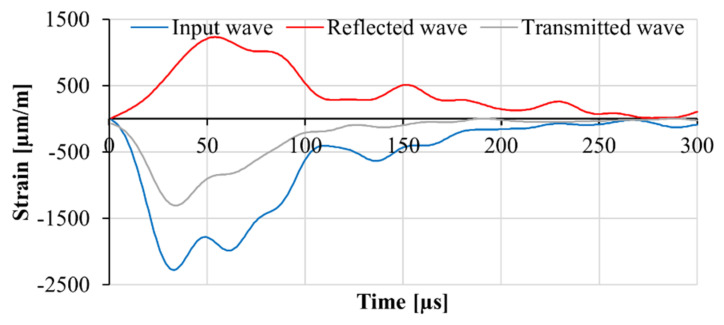
An example of the recorded input, reflected, and transmitted waves in the SHPB.

The three waves: incident, reflected and transmitted, were recorded in the middle of the respective bar using two strain gauges positioned symmetrically on the two sides of the bar’s surface. Strain gauge measurements were recorded with a frequency of 1 MHz using a SIRIUS-HS-STG data acquisition system produced by Dewesoft (Slovenia). A digital band stop filter with a range of 10 kHz to 40 kHz was used for data preprocessing. An example of the recorded input, reflected, and transmitted waves is presented in Figure 2. The wave analysis was performed according to the theory of one-dimensional wave propagation, as explained in Section 2.5. Moreover, a high-speed camera capturing 93,000 frames per second was used to monitor the specimen during the impact test. The results of the DIC were used for visualization of the failure mode in the specimen.

For both the impact and quasi-static experiments, cylindrical specimens of length 80 mm and diameter 50 mm were tested. A specimen with a length of 450 mm was also tested for all the mixtures in the split-Hopkinson pressure bar in order to calculate the dynamic Young’s modulus based on the wave propagation speed in the specimen. Furthermore, to show the influence of the specimen’s length on the failure mode in quasi-static and dynamic experiments, shorter specimens each with a length of 50 mm were also tested in the case of M1-6PE.

To produce the specimens from the cast beam, first, two blocks with lengths of 50 mm were cut from the two ends of the beams to eliminate possible end effects on the core-drilled specimens. Next, a specimen with a length of 450 mm was core-drilled from each beam. The beams were then cut into sections of 80 mm length in order to core-drill the short specimens for quasi-static and dynamic compression tests. All samples had diameters of 50 mm, the same as the bars of the SHPB, and were core-drilled longitudinally through the beams. Five specimens were tested for each parameter combination.

### 2.3. Setup for Quasi-Static Compression Tests

Quasi-static experiments were performed in a Zwick-Roell 1200 testing machine at a cross-head displacement rate of 0.08 mm/s, which resulted in a strain rate of 0.001 s^−1^ in the specimen. The force was recorded using a load-cell with a sampling frequency of 5 Hz. The deformation of the specimen was recorded utilizing DIC. A stereo camera system VIC-3D, which captures five frames per second with a resolution of 870 × 520 pixels, was used to monitor the specimens during the experiments. Subsequent digital image correlation were conducted using VIC-3D Software developed by Correlated Solutions (Irmo, SC, USA). The recordings of both the frames and the force signals were performed using the same data acquisition system. As a result, the DIC results were automatically synchronized with the force measurements.

### 2.4. Setup for Impact Shear Tests

**Figure 3:**
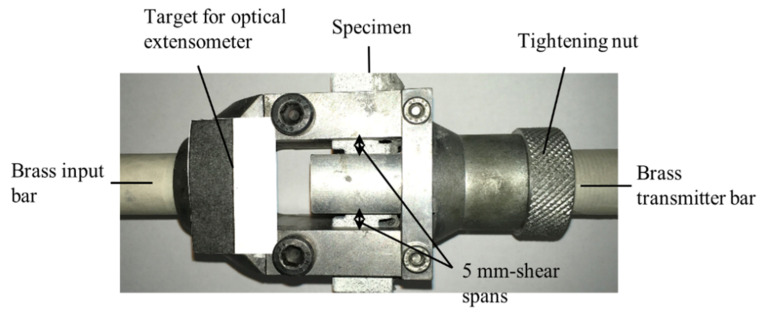
The shear adapter system installed in the SHTB.

The adapter system presented in Figure 3 was used to perform impact shear experiments in a split-Hopkinson tension bar at shear displacement rates of up to 3.5 m/s. The input and transmitter bars of the SHTB are both made of brass with a Young’s modulus of 98 GPa and having a diameter of 24 mm. A detailed description of the SHTB can be found in [23]. Through a series of calibration tests, it was shown that the conventional wave analysis according to Section 2.5 cannot provide the correct displacement of the bottom part of the adapter. The same phenomenon was observed and discussed in detail in [23] for adapters used in uniaxial tension tests. To solve the problem an optical extensometer was used to measure the displacement of the bottom adapter. Similarly, it was shown that the adapter on the transmitter bar has a minor effect on the recorded transmitted wave [19]. Thus, the transmitted wave was used to calculate both the shear stress and displacement of the upper adapter. A high-speed camera was also used to monitor the specimen during the impact shear tests. The frame rate of the camera was set to 112,500 fps. The recorded frames were subsequently used for performing a 2D DIC analysis.

**Figure 4:**
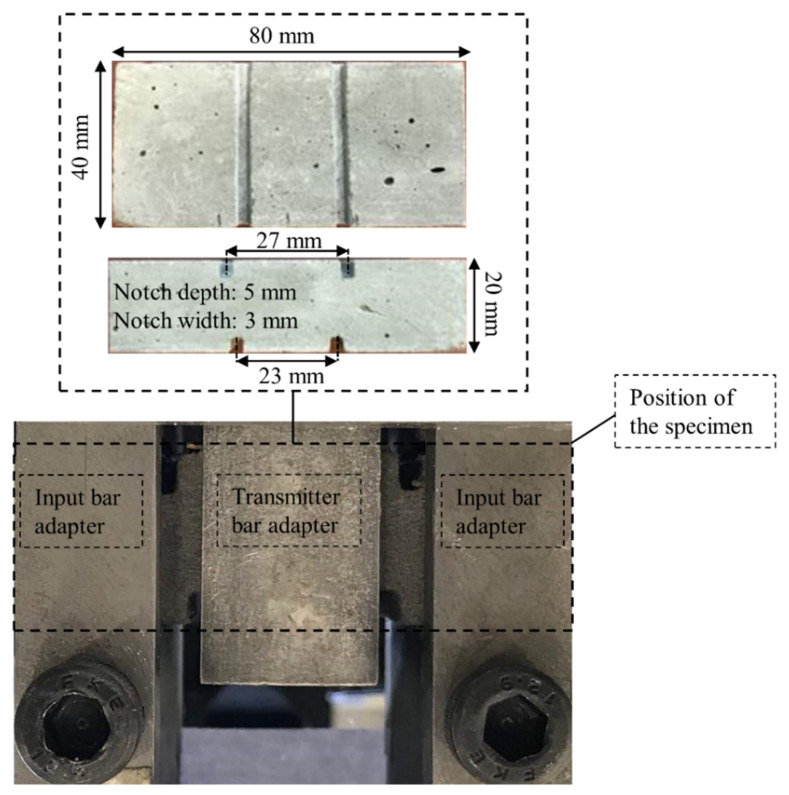
Geometrical details of the specimens tested under shear loading.

The experimental campaign consisted of impact and quasi-static shear tests on specimens made of M1-6PE SHCC and its constituent matrix. Planar specimens with dimensions of 80 mm × 40 mm × 20 mm were cut out of the cast larger prisms; see Figure 4. The same specimen geometry was used in both the quasi-static and impact experiments. Moreover, notches were cut into each specimen for a targeted concentration of shear stress in the shear band. The position of the notches and their depth are also presented in Figure 4.

**Figure 5:**
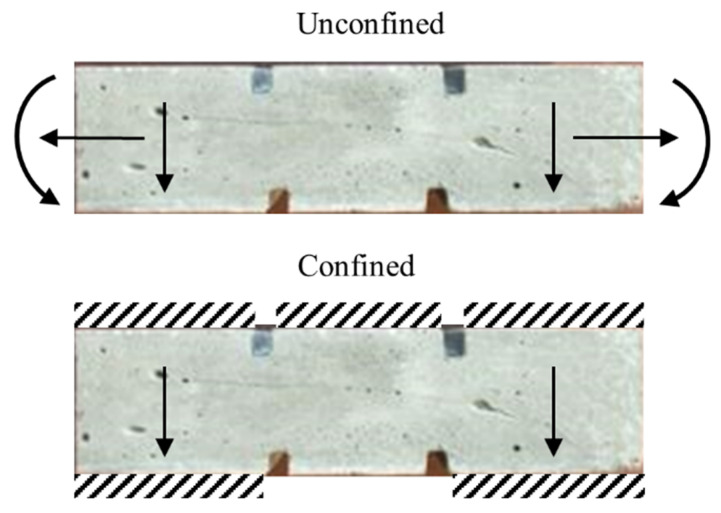
Boundary conditions of the specimen in confined and unconfined shear experiments. The arrows show the possible movement directions, and the cross-hatched areas represent glue.

Shear experiments were performed in both confined and unconfined configurations to check the influence of the specimen’s boundary conditions on the failure mode. In the unconfined experiments, the specimen is placed in the adapter, as shown in Figure 3. The two ends of the specimen can expand and rotate in this configuration. In the confined experiments, the specimen is glued to the side grips of the input bar adapter. The upper side of the specimen is also glued to the adapter part on the transmitter bar. Figure 5 shows the boundary conditions of the specimen in both testing configurations. For each parameter combination, four specimens were tested.

### 2.5. Setup for Quasi-Static Shear Tests

The same adapter as that used in the impact tests was used in the quasi-static experiments. In these experiments, the bars screwed to the adapters were clamped in an Instron 8501 servo-hydraulic testing machine. The displacement rate in the quasi-static experiments was 0.05 mm/s, which resulted in a shear strain rate of 0.01 s^−1^ in the symmetrical 5 mm shear bands of the specimen; see Figure 4. The displacement was measured using an optical extensometer.

### 2.6. Wave Analysis Procedure for Split-Hopkinson Bar Tests

The wave analysis in both split-Hopkinson bars was performed according to the theory of one-dimensional wave propagation [24]. The stresses at both ends of the specimen are compared to check stress uniformity in the specimen, and the average stress is considered for the evaluation of the material properties. Equations (1) and (2) are used to calculate the forces at each end of the specimen based on the input, reflected, and transmitted signals ($\varepsilon_{I},\;\varepsilon_{R},\;\varepsilon_{T}$). In these equations, E and A are the Young’s modulus and the cross-sectional area of the bars, respectively. The displacement at the two ends of the specimen is calculated based on Equations (3) and (4), with C being the elastic wave velocity of the input and the transmitter bars. Note that for shear tests, measures derived by Equations (3) and (4) correspond to the displacement of the adapters on the input and transmitter bar, respectively. The stress in the specimen is calculated using Equation (5), where $A_{s}$ is the cross-sectional area of the specimen. The strain in the specimen is found using Equation (6), where $L_{s}$ is the length of the specimen, or in the case of shear tests the width of the shear span. The strain rate is calculated using Equation (7).

(1) $$F_{i}\left(t\right)=EA\left(\varepsilon_{I}\left(t\right)+\varepsilon_{R}\left(t\right)\right)$$

(2) $$F_{t}\left(t\right)=EA\varepsilon_{T}\left(t\right)$$

(3) $$\delta_{i}\left(t\right)=C\int_{0}^{t}\left(\varepsilon_{I}\left(t\right)-\varepsilon_{R}\left(t\right)\right)dt$$

(4) $$\delta_{t}\left(t\right)=C\int_{0}^{t}\varepsilon_{T}\left(t\right)dt$$

(5) $$\sigma \left(t\right)=\frac{F_{i}\left(t\right)+F_{t}\left(t\right)}{2A_{s}}$$

(6) $$\varepsilon \left(t\right)=\frac{\delta_{i}\left(t\right)-\delta_{t}\left(t\right)}{L_{s}}$$

(7) $$\dot{\varepsilon }\left(t\right)=\frac{\left(\varepsilon_{I}\left(t\right)-\varepsilon_{R}\left(t\right)-\varepsilon_{T}\left(t\right)\right)C}{L_{s}}$$

In order to obtain the dynamic Young’s modulus based on the wave propagation velocity in the long specimens, Equation (8) is used. In this equation, ρ is density, and C is the wave propagation velocity in the specimen found based on the time needed for the wave to pass through the specimen.

(8) $$E=C^{2}\rho$$

## 3. Results of the Compression Tests

### 3.1. Results of the Quasi-Static Compression Tests

**Figure 6:**
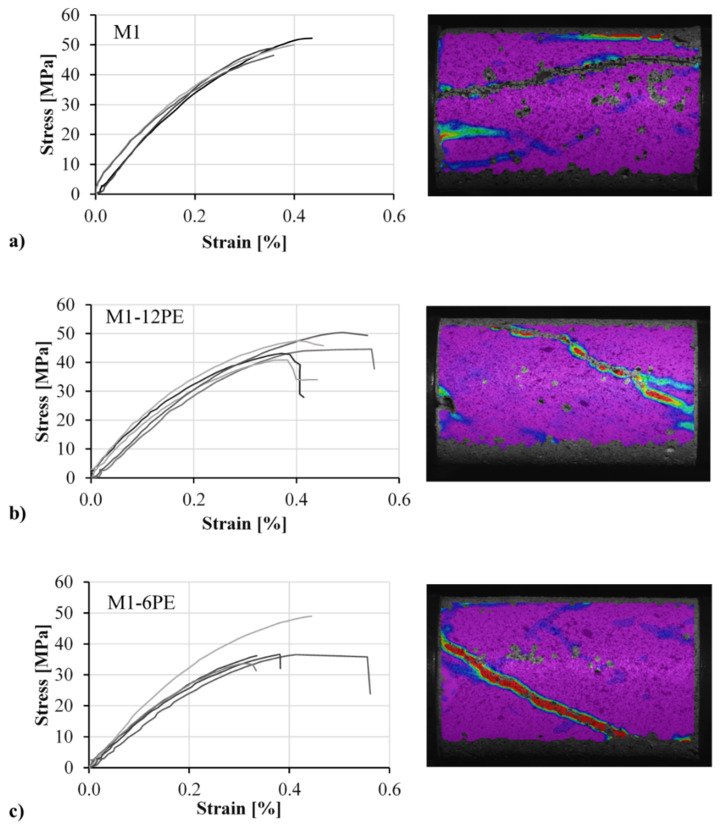
Stress-strain curves and corresponding typical failure modes (digital image correlation (DIC) images) of specimens obtained under quasi-static loading for the mixtures: (**a**) M1, (**b**) M1-12PE, and (**c**) M1-6PE. The specimen’s length was 80 mm, and the diameter was 50 mm.

Stress-strain relations of the three mixes and their typical final failure patterns are presented in Figure 6. The stress was calculated based on the force recorded by the load cell of the testing machine, and the strain was calculated based on the DIC results. For this purpose two virtual gauges were placed on two sides of the displacement field calculated by means of DIC. Average deformation of the two virtual gauges was used to calculate strain. Note that the stress-strain curves are presented up to the peak compressive stress only. After the sudden failure of the specimen, the vibration of the load cell impaired the recording of the force. Furthermore, in all cases, the final failure surface was inclined due to the shear induced by the friction between the specimen and the loading plates.

A comparison of the results obtained for the three mixtures under investigation revealed a slight reduction in strength through adding the fibers to the cementitious matrix; see Table 3. While the non-reinforced matrix M1 demonstrated a compressive strength of 48.6 MPa, the two SHCC mixtures M1-12PE and M1-6PE showed compressive strengths of 45.3 MPa and 35.9 MPa, respectively. This can be traced back to the higher porosity of SHCC since some air can be trapped by fine and dense lattices of fibers. A similar effect was observed also in previous studies on fiber-reinforced concrete [25]. Furthermore, the fact of HDPE fibers establishing a weak frictional bond to the matrix makes them flaws in the matrix. Put another way, the addition of fibers introduces more voids and flaws into the matrix, resulting in a reduction in compressive strength. This seems to be more pronounced for the specimens containing the 6 mm fibers. Note that the reduction of fiber length from 12 mm to 6 mm means a doubling of the number of fibers, obviously amplifying the effects mentioned above. Moreover, considering the production method of the specimens, it can be assumed that the majority of fibers were oriented in the direction of loading, which is unfavorable with respect to crack control. Thus, presumably such fibers cannot hinder the development of cracks, but rather trigger the cracking process [26].

Generally, two effects, namely the role of the fibers as flaws and porosity “drivers” in the matrix and their action as reinforcement in preventing damage growth, counteract each other in influencing the compressive strength of the SHCC. However, the results obtained indicate that the hydrophobic fibers have a more substantial influence than the flaws, as both SHCC mixtures showed lower compressive strength than the plain matrix. In the case of strain capacity, considering the scattering of the results, it can be concluded that there was no significant influence due to the addition of fibers. This result is in contrast to an earlier investigation at the TU Dresden, where a much higher SHCC compressive strain capacity was observed [27]. However, in that case SHCC made with PVA fibers was investigated, and the loading occurred perpendicular to the direction of casting. The Young’s modulus exhibits a trend similar to that observed for compressive strength. The addition of fibers slightly reduced the stiffness of the SHCC since the hydrophobic fibers act as voids inside the matrix and additionally induce higher porosity through the entrapped air.

Young’s modulus was calculated based on the slope of the stress-strain curves up to 40% of the maximum stress, where the behavior was still linear-elastic. In this range of stresses, it was assumed that the damage and microcrack growth in the specimen were still negligible, so the crack arrest action of the fibers to that point had not yet been activated.

**Figure 7:**
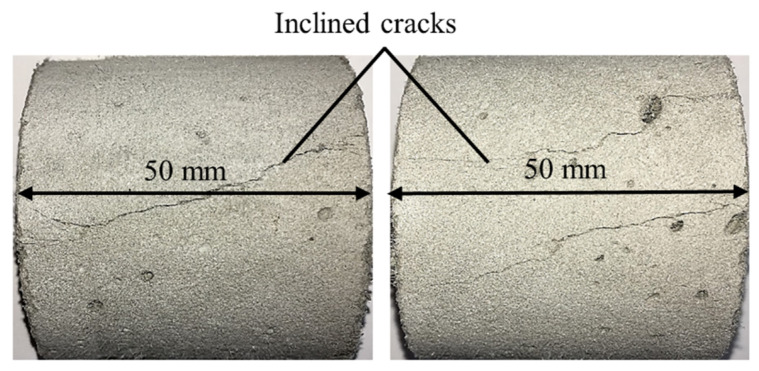
Two examples of crack pattern on the surfaces of M1-6PE specimens with a length of 50 mm tested under quasi-static compression.

Shorter specimens with a length of 50 mm made of M1-6PE were also tested under quasi-static loading. The purpose of these experiments was to investigate the failure mode in the specimen. Similar to the longer specimens, these specimens exhibited inclined shear cracks when they failed under a quasi-static compressive load. Figure 7 shows examples of failure patterns visible on the surfaces of the tested specimens. The same size of the specimen was also tested in SHPB to show the change in the failure pattern of short specimens under impact compressive loading.

### 3.2. Results of the Impact Compression Tests

In the dynamic compression testing of brittle and quasi-brittle materials performed in a split-Hopkinson pressure bar, the influence of friction between the ends of the specimen and the bars was emphasized by several researchers [28,29]. The combined effects of friction and radial inertia, generated by acceleration applied to the specimen, provide lateral confinement for the inner core of the specimen [14]. The generated multiaxial pressure causes an increase in the compressive strength of the specimen, which is not genuinely related to the rate sensitivity of the material.

**Figure 8:**
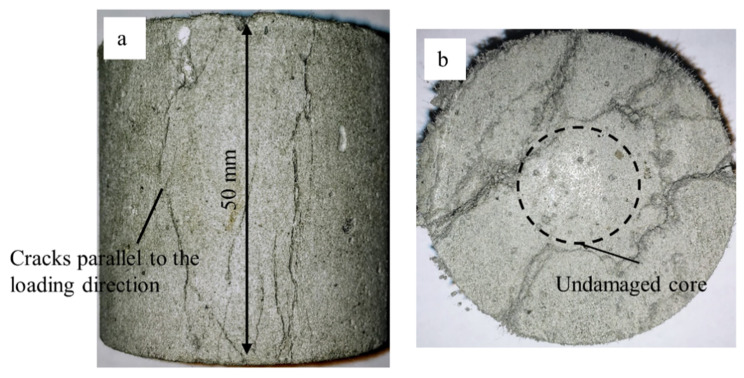
Failure mode in a M1-6PE specimen tested in the SHPB: (**a**) cracks parallel to the loading direction, (**b**) middle cross-section of the specimen.

The aforementioned mechanisms not only lead to an overestimation of the compressive strength of the cement-based materials due to their confining stress-dependent behavior but also cause different failure modes in the experiments performed in split-Hopkinson pressure bars as compared with quasi-static experiments. Figure 8 shows an example of a specimen made of M1-6PE with a length of 50 mm tested in the split-Hopkinson pressure bar. There is a clear distinction in the failure modes of the specimens in Figure 7 and Figure 8 tested under quasi-static and impact compression, respectively. While the quasi-static experiments on cylinders with a length of 50 mm show an inclined shear crack, the dynamic experiments mostly show damage on the outer surface in the form of cracks parallel to the loading direction and a less damaged inner core of the specimen, which can be traced back to the higher strength of the material due to the confinement [15]. The differences in the stress state and the failure mode in quasi-static and impact experiments make it difficult to compare the compressive strength under these two loading conditions.

**Figure 9:**
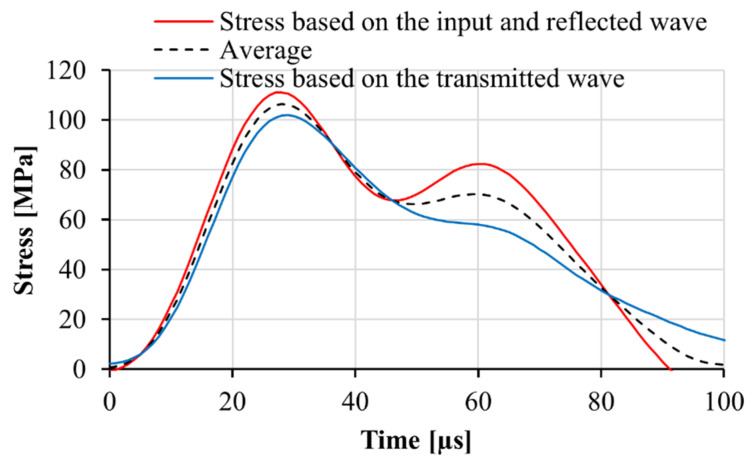
Stress-time histories in a matrix M1 specimen recorded at the specimen-input bar and specimen-transmitter bar interfaces. The specimen’s length was 80 mm.

To obtain similar stress states and failure modes in impact and in quasi-static experiments, specimens with identical geometry, length of 80 mm and diameter of 50 mm, were used for both types of test. The selected geometry resulted in comparable failure modes under the two loading regimes. However, the similar failure modes were achieved at the cost of reducing the stress uniformity along the specimen tested in the SHPB. The specimens with a length of 80 mm show slight deviations of the stresses calculated on two sides of the specimen connected to the input bar and transmitter bar, respectively. An example of the stresses calculated on both sides of the specimen is presented in Figure 9.

**Figure 10:**
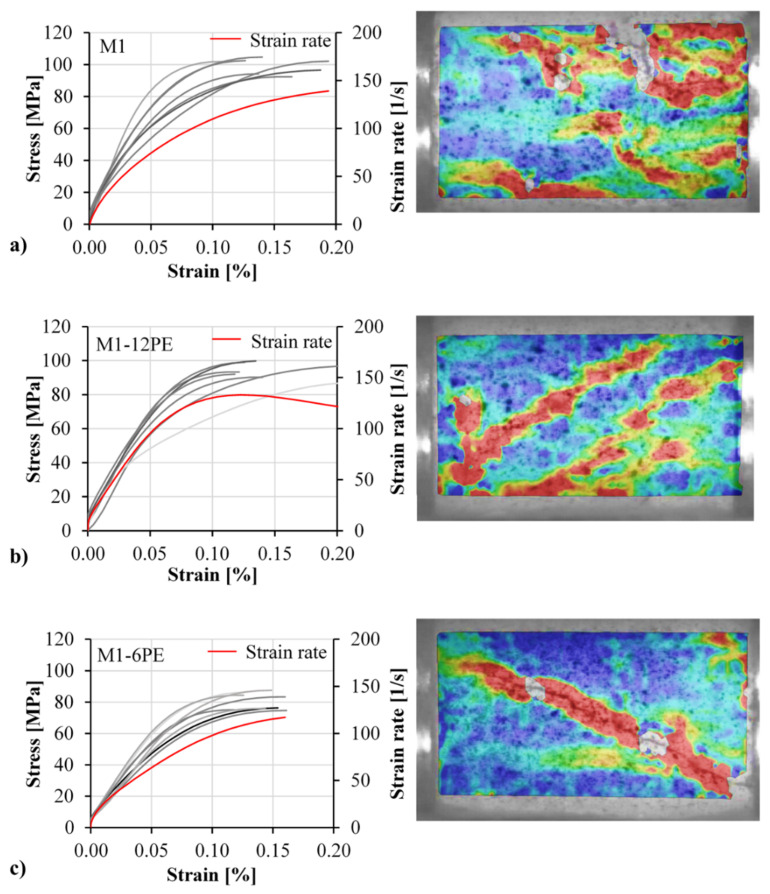
Stress-strain curves and the corresponding typical failure modes of the specimens (DIC images) obtained in SHPB for mixtures: (**a**) M1, (**b**) M1-12PE and (**c**) M1-6PE.

As in the quasi-static tests, the unreinforced matrix shows the highest compressive strength, followed by M1-12PE and M1-6PE with compressive strengths of 99.2 MPa, 94.1 MPa, and 81 MPa, respectively. Moreover, a very similar trend in the results can be observed. While M1-12PE showed compressive strength slightly lower than that of the unreinforced matrix, M1-6PE yielded a significantly lower compressive strength. The average compressive strength of the three mixtures and the corresponding dynamic increase factor (DIF) are given in Table 4. The stress-strain diagrams obtained in the split-Hopkinson bar experiments are presented in Figure 10. A representative strain rate curve is also provided for each mixture.

**Figure 11:**
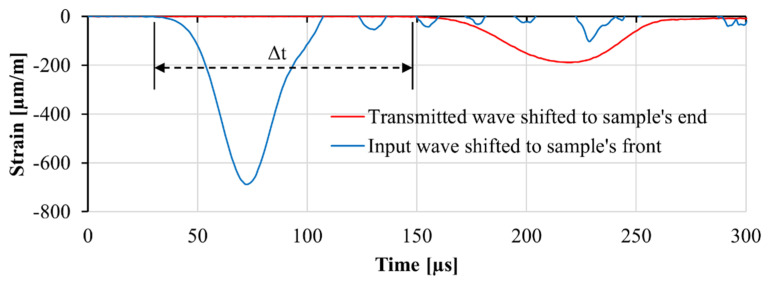
An example of the input wave and transmitted wave shifted respectively to the front and backside of the long specimen. Δt denotes the time needed for the wave to travel through the specimen.

The specimens tested in a split-Hopkinson bar are subject to a pronouncedly non-uniform stress distribution at the initial part of the loading wave. As a result, Young’s modulus cannot be calculated from the obtained stress-strain curves. Therefore, to obtain Young’s modulus of the three mixtures under investigation at a high strain rate, specimens with a length of 450 mm were tested in the same split-Hopkinson pressure bar [30]. In these experiments, the amplitude of the wave was reduced to prevent any damage in the specimen through passage of the stress wave. The time needed for the wave to travel from one side of the specimen to the other side was used to calculate Young’s modulus of the specimen using Equation (8). For this purpose, the input wave was shifted to the end of the input bar, and the transmitted wave was shifted to the beginning of the transmitter bar. The difference between the rise time of the two signals shows the time needed for the wave to travel through the specimen. The wave propagation speed and Young’s modulus were then calculated accordingly. An example of the recorded signals in the case of the M1 matrix is presented in Figure 11. In this figure, the decay of the wave as it travels from the strain gauge on the input bar to the one on the transmitter bar can be observed. The change in the amplitude of the wave was firstly caused by the impedance mismatch between the specimen and the bar, which limits the transmission of the input wave to the specimen. Secondly, the dispersion of the wave as it travels through the input bars, transmitter bars, and the specimen itself results in a reduction in its amplitude. It should be noted that in these experiments, an input wave shorter than the length of the specimen was used. Thus, the transmitted wave was not affected by the reverberation of the waves within the specimen.

The values of Young’s modulus as obtained are presented in Table 4. There is no standard deviation provided in this case since only one specimen was tested per mixture due to technical difficulties in core drilling the long specimens. In case of M1-12PE, a slight misalignment of the long specimen in between the bars of SHPB led to the failure of the specimen. In case of the matrix M1 and SHCC M1-6PE, the values obtained were slightly higher than the quasi-static ones. A similar trend was observed in [21], where comparable materials were tested using a tensile stress wave in a split-Hopkinson tension bar.

## 4. Results of the Shear Tests

### 4.1. Results of the Quasi-Static Shear Tests

**Figure 12:**
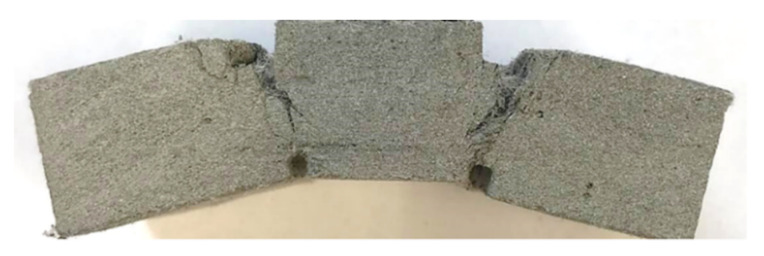
Flexural failure in an unconfined specimen tested under quasi-static shear loading.

The experimental series started with unconfined shear tests; cf. Figure 5. Despite the narrow shear span and the notch in the specimen, a flexural failure mode was obtained; see Figure 12. Hence, it was concluded that the lateral and rotational movements of the two sides of the specimen need to be constrained. Once the two sides of the specimen were fixed in the adapter, shear failure of the specimens was obtained.

**Figure 13:**
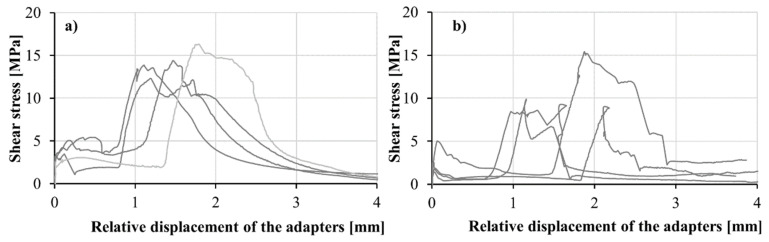
Shear stress vs. displacement curves obtained in the confined quasi-static tests on (**a**) M1-6PE SHCC, and on (**b**) plain M1 matrix.

The shear behavior of the confined M1-6PE and M1 specimens under quasi-static loading are presented in Figure 13. Due to the technical limitations of the optical extensometer, the displacements were measured at a certain distance from the shear span of the specimen; see the position of the optical target and the specimen in Figure 3. Thus, in these experiments, the relative displacement of the two sides of the adapter was likely affected by deformation in the adapter parts. As a result, the shear strain cannot be discussed here, and the shear stress is plotted with respect to the relative displacement of the two parts of the adapter. The shear stress in the diagram is calculated by dividing the measured force by the cross-section area of the two shear bands.

**Figure 14:**
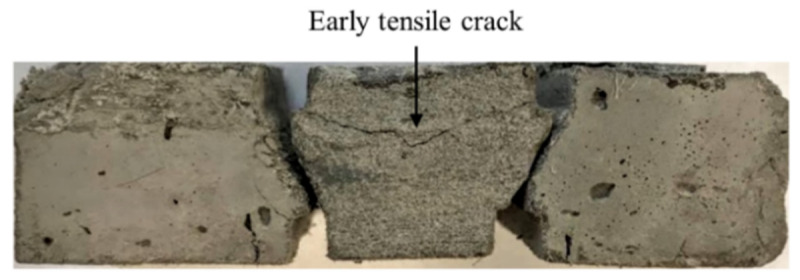
An example of the initial tension crack formed in the confined specimens tested under quasi-static shear loading.

At the beginning of the curves obtained for both materials, a rise in shear stress is observed, corresponding to the elastic behavior of the specimen. Due to the formation of a horizontal crack on the top of the specimen, where it is glued to the adapter on the transmitter bar, the increase in stress terminates. Figure 14 shows the early-stage tension crack in a confined specimen tested under quasi-static shear loading. After formation of the tension crack, due to a reduction in the system’s stiffness, stress remains constant as the tensile crack, formed accordingly, opens. However, once the relaxation phase ends, the shear stress starts to rise again. At this stage, mode II diagonal cracks form in the specimen in the paths, predefined by the notches in the sheared zone. The stress starts to decrease after the diagonal cracks are formed. Multiple cracking under shear loading did not occur due to the considerably lower shear load-bearing capacity of the fibers bridging the crack in comparison to that of the matrix. The results of the confined quasi-static shear experiments on M1 matrix and M1-6PE SHCC are summarized in Table 5 together with the results of the impact tests. The average shear strength of the plain matrix was 10.8 MPa, while that of matrix reached 14.0 MPa. A more detailed discussion on the fracture process of the specimen is provided for the case of impact loading, for which the results of the high-speed camera recording and subsequent DIC analysis are also presented. In the quasi-static experiments, no synchronized optical measurement was performed.

### 4.2. Results of the Impact Shear Tests

**Figure 15:**
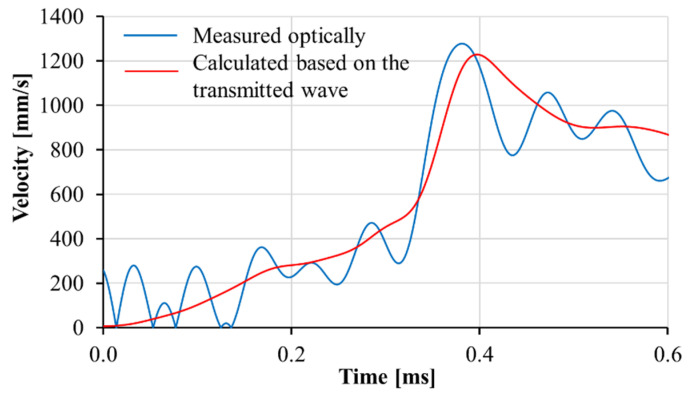
Results of a calibration test showing agreement between the velocity of the adapter measured optically and that calculated based on the transmitted wave.

**Figure 16:**
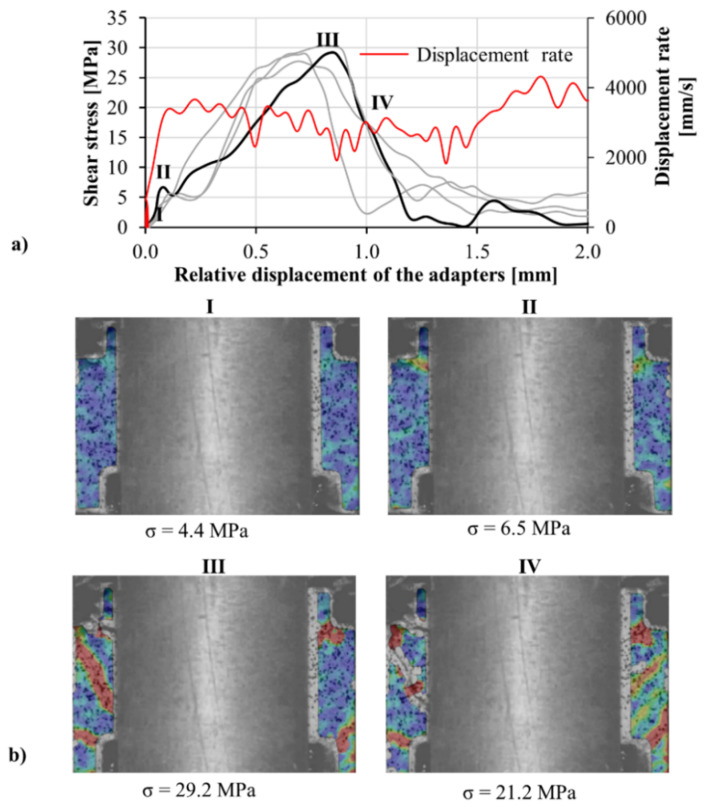
(**a**) Shear stress versus displacement curves obtained in the confined impact shear tests on M1-6PE SHCC, and (**b**) a representative DIC sequence showing the first principal strain field in the shear zones for various stress levels corresponding to the thicker black curve.

**Figure 17:**
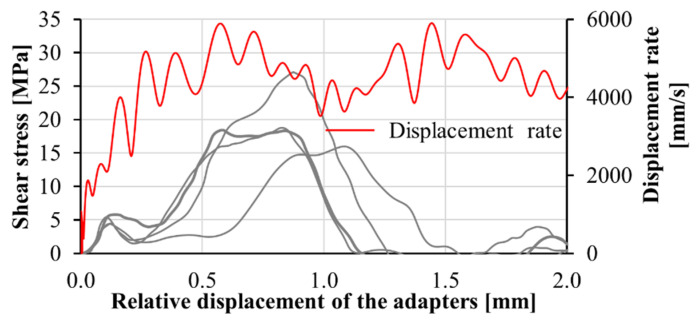
Shear stress versus displacement curves obtained in the confined impact shear tests on M1 matrix.

Calibration procedures were performed to confirm the reliability of the transmitted wave in finding the displacement of the adapter on the transmitter bar. The velocity as measured by optical extensometer was compared to that calculated based on the wave analysis using the first derivative of Equation (4) with respect to time. The acceptable agreement between the two methods shows the reliability of the transmitted signal; see Figure 15. In these experiments, the stress was calculated based on the transmitted wave. To calculate the relative displacement of the adapter parts, displacement of the part connected to the input bar was measured optically, and the displacement of the part connected to the transmitter bar was derived by Equation (4) using the recorded transmitted wave. The shear behavior of the M1-6PE and M1 specimens obtained from confined impact experiments are presented in Figure 16 and Figure 17, respectively.

In both Figure 16 and Figure 17, a sudden drop in the shear stress is observed at the beginning of the curve after the initial rise of the stress. The tension crack on the top side of the specimen, observable in frame II in Figure 16, is the reason behind this drop. The crack is formed due to the tensile stress generated close to the region of the specimen, which is glued to the transmitter bar adapter. Note that the middle-top surface of the specimen was glued to the adapter to prevent the formation of flexural cracks. Once the early tensile crack is opened, the shear stress starts to rise rapidly. The progressive increase in shear stress stops with the formation of inclined cracks, as observed in frame III in Figure 16. Unlike the tensile crack, the inclined cracks are related to the shear load applied to the specimen. The applied shear load rotates the direction of first principal stress. As a result, the inclined cracks form perpendicular to the direction of the first principal stress under tension in both shear spans. Once the inclined cracks are formed in the cement-based matrix, the shear stress starts to decrease rapidly in the case of the plain matrix M1. The reduction in the shear stress is more gradual for the SHCC specimens than for that of the matrix since the shear load is transferred to the fibers bridging the cracks. The results of impact experiments are summarized in Table 5. Similar to the quasi-static tests, under impact loading, the shear strength of the SHCC was higher than that of the plain matrix. The favorable alignment of fibers in the longitudinal direction of the specimen can justify the higher shear strength of the SHCC.

**Figure 18:**
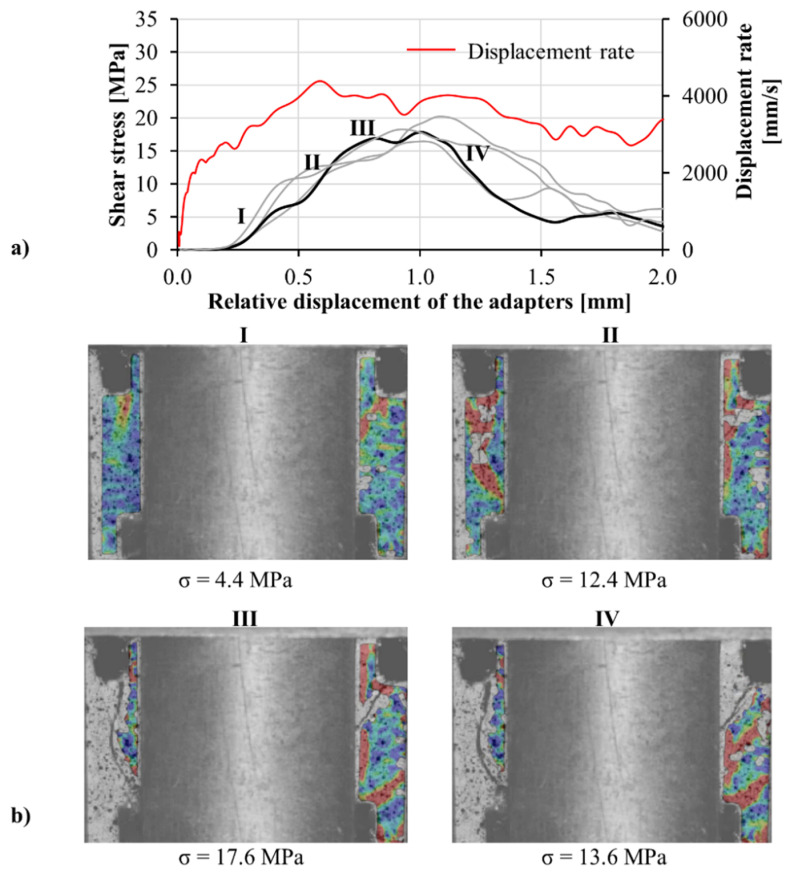
(**a**) Shear stress versus displacement curves obtained in the unconfined impact shear tests on M1-6PE SHCC, and (**b**) a representative DIC sequence showing the first principal strain field in the shear zones for various stress levels corresponding to the thicker black curve.

The important role of the boundary conditions in achieving shear failure in the specimen was shown based on the results of quasi-static tests. However, for the sake of comparison, the results of unconfined impact shear experiments on M1-6PE specimens are provided in Figure 18. The representative DIC analysis shows that unlike the confined case, the specimen is substantially loaded by the bending moment. The rotation of the two sides of the specimen, as can be observed in the DIC sequence, creates a bending moment in the specimen. As a result, tension cracks are formed on both sides of the notches.

## 5. Conclusions

The findings of the impact compressive experiments can be outlined as follows:

- The length of the specimen can significantly influence the failure mode under dynamic loading. The short specimens failed differently under quasi-static and dynamic loading. By increasing the length of the specimen, similar failure modes consisting of diagonal shear cracks were observed in both loading regimes. The similarity of the failure modes made a comparison between the obtained compressive strengths meaningful. The highest dynamic increase factor (DIF), equal to 2.3, was obtained for M1-6PE. Moreover, the results indicate a reduction in compressive strength when adding HDPE fibers to the plain M1 matrix.
- The values of Young’s modulus for M1 and M1-6PE obtained by measuring the wave propagation speed in the long specimens were comparable to those obtained in tension experiments using the same method. In both cases, a slight increase was observed under dynamic loading.
- The predominant orientation of the fibers in the SHCC specimens was parallel to the loading direction, which limits the effectiveness of the fibers as crack control constituents. The lower compressive strength of SHCC compared to the plain matrix can be traced back both to the unfavorable alignment of the fibers and to higher porosity of SHCC. Further investigations on specimens with different predominant fiber orientations are required.

The results obtained point out possible influences of specimen geometry and failure mode, which should be considered when analyzing the results of impact compression tests. Moreover, the reduction in compressive strength due to the addition of HDPE fibers to the cement-based matrix indicates the importance of fiber orientation and fiber type in tailoring the behavior of SHCC for impact loading.

The shear experiments performed on M1-6PE showed that:

- The designed adapter can be used in performing impact shear experiments in the SHTB. However, obtaining shear failure is only possible by applying lateral and rotational constraints on both sides of the specimen. The DIC analysis revealed the influence of an early-stage tensile crack in the specimen on the calculated shear stress. The tensile crack was formed in the middle of the specimen and outside the sheared span of the specimen.
- Although the M1-6PE SHCC showed lower compressive strength when compared to the plain matrix, under shear loading the shear strength of the SHCC was considerably higher than that of the constituent matrix.

The results presented show the possibility of characterizing the behavior of SHCC under impact shear loading in SHTB. These results can be used in optimizing the shear behavior of SHCC, which is of great importance with respect to its application as a strengthening layer on the impact-facing side of existing structures. High energy absorption capacity and proper distribution of the impact load to the substrate structure is crucial in such an application scenario.

## Figures and Tables

**Table 1:** Composition of the matrix and strain-hardening cement-based composite (SHCC) under investigation.

M1-6PE and M1-12PE	[kg/m^3^]	Producer
CEM I 42.5R-HS	505	SCHWENK
Fly ash Steament H4	621	STEAG
Quartz sand 0.06–0.2 mm	536	Strobel
Viscosity-modifying agent	4.8	Sika
Water	338	
Superplasticizer Glenium ACE 30	10	BASF
High-density polyethylene (HDPE) fiber (length of 6 or 12 mm)	20	DSM

**Table 2:** Physical and mechanical properties of HDPE fibers.

Density [g/cm^3^]	0.97
Diameter [µm]	18
Tensile strength [MPa]	2500
Young’s modulus [GPa]	80
Strain capacity [%]	4

**Table 3:** Mechanical properties of the matrix and SHCC mixtures obtained in quasi-static compression tests (standard deviations are given in parentheses).

Mechanical Properties	Matrix M1	M1-12PE	M1-6PE
Compressive strength [MPa]	48.6 (1.4)	45.3 (3.7)	35.9 (1.3)
Strain capacity	0.38 (0.04)	0.43 (0.08)	0.35 (0.06)
Young’s modulus [GPa]	19.7 (1.7)	17.7 (1.9)	15.7 (1.8)

**Table 4:** Mechanical properties of the matrix and SHCC mixtures obtained in impact compression tests (standard deviations are given in parentheses).

Mechanical Properties	Matrix M1	M1-12PE	M1-6PE
Compressive strength [MPa]	99.2 (4.5)	94.1 (4.7)	81 (5.1)
Dynamic increase factor (DIF) of compressive strength	2.0	2.1	2.3
Strain capacity [%]	0.15 (0.03)	0.14 (0.04)	0.15 (0.01)
Young’s modulus [GPa]	24.0	-	22.3
Strain rate at failure [s^−1^]	111 (14)	109 (20)	113 (9)

**Table 5:** Quasi-static and impact shear strengths of M1-6PE and its non-reinforced matrix (standard deviations are given in parentheses).

Mechanical Properties	Matrix M1	M1-6PE
Quasi-static shear strength [MPa]	10.8 (3.1)	14 (1.5)
Impact shear strength [MPa]	19.5 (3.8)	28.5 (1.6)
DIF	1.8	2.0
